# Recent heart rate history affects QT interval duration in atrial fibrillation

**DOI:** 10.1371/journal.pone.0172962

**Published:** 2017-03-08

**Authors:** Fady S. Riad, Eathar Razak, Samir Saba, Alaa Shalaby, Jan Nemec

**Affiliations:** 1 Department of Internal Medicine, The University of Pittsburgh Medical Center, Pittsburgh, Pennsylvania, United States of America; 2 Department of Cardiology, VA Pittsburgh Healthcare System, Pittsburgh, Pennsylvania, United States of America; 3 Heart and Vascular Institute, The University of Pittsburgh Medical Center, Pittsburgh, Pennsylvania, United States of America; University of Minnesota, UNITED STATES

## Abstract

QT interval prolongation is associated with a risk of polymorphic ventricular tachycardia. QT interval shortens with increasing heart rate and correction for this effect is necessary for meaningful QT interval assessment. We aim to improve current methods of correcting the QT interval during atrial fibrillation (AF). Digitized Holter recordings were analyzed from patients with AF. Models of QT interval dependence on RR intervals were tested by sorting the beats into 20 bins based on corrected RR interval and assessing ST-T variability within the bins. Signal-averaging within bins was performed to determine QT/RR dependence. Data from 30 patients (29 men, 69.3±7.3 years) were evaluated. QT behavior in AF is well described by a linear function (slope ~0.19) of steady-state corrected RR interval. Corrected RR is calculated as a combination of an exponential weight function with time-constant of 2 minutes and a smaller “immediate response” component (weight ~ 0.18). This model performs significantly (p<0.0001) better than models based on instantaneous RR interval only including Bazett and Fridericia. It also outperforms models based on shorter time-constants and other previously proposed models. This model may improve detection of repolarization delay in AF. QT response to heart rate changes in AF is similar to previously published QT dynamics during atrial pacing and in sinus rhythm.

## Introduction

QT interval prolongation reflects an increased duration of the ventricular action potential. It is associated with a risk of *torsade de pointes* (TdP) and sudden cardiac death in individual patients [1,2] and with total and cardiovascular mortality in epidemiological studies [3–7].

QT interval duration is strongly affected by heart rate (HR) and several formulas have been proposed for HR correction of the QT interval. Most of them perform better than the original Bazett formula, which is still frequently used at the bedside. Nevertheless, there are substantial differences even among individual healthy subjects with respect to the dependence of QT interval on HR, putting the concept of a universal QT correction formula into question [8]. Moreover, it is recognized that QT interval changes follow HR changes with some delay, resulting in the appearance of “hysteresis” in QT/RR interval plots [9].

We have previously analyzed QT interval response to sudden change in atrial pacing rate in otherwise healthy patients undergoing radiofrequency ablation of supraventricular tachycardia [10]. These results demonstrated that the steady-state QT interval depends on RR interval (inverse of the HR) as a linear function, and that a new steady-state QT value after HR change is attained with a time-constant of approximately 60–90 seconds. When the beat-to-beat RR interval changes are large, an “immediate response,” which occurs without delay and accounts for approximately 20% of overall QT response, can also be resolved. QT interval accommodation during sinus rhythm (SR) exhibits similar dynamics [11,12].

Compared to SR, less is known about QT interval dynamics in atrial fibrillation (AF). This is in part due to the high RR interval variability present in most patients in AF, which makes the selection of the “correct” RR interval to use in the QT correction formulas problematic. On the other hand, detection of repolarization delay during AF is of substantial clinical significance, for example during initiation of treatment with class III antiarrhythmic agents. Several methods have been suggested to deal with the problem [13–18]. We sought to determine if the exponential weight models with an immediate response component derived from SR and atrial pacing data also provide a consistent description of QT dynamics in AF. We found that such models outperform current models used in clinical practice such as Bazett and Fridericia.

## Methods

We performed a retrospective review of ambulatory ECG tracings obtained at the VA Pittsburgh Healthcare System in 2013 and 2014. All the ambulatory ECG studies were obtained with the GE Seer Light recorder and exported in the digital format with the MARS 7.2 Holter analysis system (GE Health). The monitoring duration was 24 hours, the sampling frequency was 125 Hz and the sampling precision was 20 μV. Tracings with at least 6 hours of AF during the monitoring period were considered for analysis. Subjects with cardiac rhythm management devices or marked changes in QRS morphology during the recording (e.g. frequent intermittent aberrancy) were excluded. This study was approved by the VA Pittsburgh Healthcare System IRB. The digitized tracings were edited and analyzed using a custom-made software created in C++ (Microsoft Visual Studio) by one of the investigators (JN). Briefly, the channel with the best signal quality was selected and automatically annotated with respect to R wave peaks. Subsequently, the signal was low-pass filtered at 20 Hz (3-pole Butterworth filter) and manually reviewed by 2 consecutive reviewers (FR, JN) to eliminate data segments that were unsuitable because of noise, baseline wander, incorrect automatic R wave detection, ventricular ectopy, or other reasons. Patients with < 5,000 analyzable beats were excluded from analysis. An example of an acceptable data segment is shown in **S1 Fig**.

It is more difficult to obtain reliable direct QT interval measurements in AF than in sinus rhythm, primarily because of the superimposition of the atrial fibrillatory wavelets on the terminal portion of the T wave. For this reason, we used a combination of binning and signal averaging for most of the signal analysis. This approach is based on the assumption that the true underlying changes in ST-T segment morphology (which determines QT interval duration) are independent of “noise,” which in this case includes the fibrillatory atrial activity. Making this assumption, we judged the model fit by the magnitude of squared differences between the signals within the 20 bins. This intra-bin variability contains both the “noise”–which cannot be reduced by the model–and the differences between the “true” ST-T segments within the individual bins. A good model will sort similar ST-T segments into the same bins, minimizing the intra-bin variability. Although different subjects of course have quite different levels of noise in their Holter recordings, this can be addressed by using differences between intra-bin variability among models within a given patient as the measure of model fit, eliminating the patient-specific differences in noise level. We used this method to compare the performance different classes of QT correction models as well as different models within a class. Because no “gold standard” exists for measuring QT interval in afib, we define the gold standard as the model which minimizes intra-bin signal variability. We then used signal averaging within each bin to eliminate noise and measure the actual QT interval. This allows us to compare QT/RR regression between models.

In addition to this approach, we have also performed manual QT interval measurements using electronic calipers, selecting 20 good-quality QT intervals from each-subject. Here, we used the SD of QTc values derived from the manual measurements by individual models, as the measure of fit. This is very similar to using the manual measurements as a direct gold-standard, with respect to model-predicted QT values.

The individual steps involved in the methods above are delineated below.

### Optimal model selection in individual subjects

Each recording was analyzed using the following steps:

1/ after manual editing, all heartbeats preceded by at least 180 s of uninterrupted data of acceptable quality were divided into 20 bins containing an equal number of beats. The beats were sorted into ventiles (i.e. bins containing 5% of the beats each) based on the duration of the preceding RR interval (RR_0_) and signal-averaging was performed in each bin. This model is referred to as **M0** below. In this and the other models, the number of analyzed beats was rounded to nearest lower multiple of 20 by removing 0 to 19 beats from the end of the list of analyzable beats. This was done to assure the same number of beats in each bin.

2/ the sum of squared differences (SSQ) between the signal values corresponding to each beat and signal values of the signal-averaged complex from the appropriate bin[14] was then calculated for each bin. The summation was performed over the repolarization window starting 80 ms after R wave peak and ending 550 ms after R wave peak but at least 100 ms before the next R wave peak.

3/ this process was repeated for the class of exponential models with time constants of 120, 60, and 30 s. In these models (labeled **Mexp120**, **Mexp60** etc.), the heartbeats were sorted into ventiles based not on increasing RR_0_ (the RR interval immediately preceding the analyzed beat), but rather on the corrected RR Interval (RRc), which is calculated as a function of the RR intervals over the preceding 3 minutes. This weight function incorporates the exponential decline of the influence of past RR intervals as well as the “immediate response” of QT interval to RR_0_. The coefficient of immediate response was tested for all values between 0 and 1 in steps of 0.01 for each model. More formally, the corrected RR interval was calculated as

$$RRc=IR.{RR}_{0}+(1-IR).\frac{\sum_{i=-1}^{-\infty }{RR}_{i}.w_{i}}{\sum_{i=-1}^{-\infty }w_{i}}$$ (1)

where *IR* is the immediate response coefficient and *w*_*i*_ is the weight assigned to the *i*^th^ RR interval preceding RR_0_. For the exponential models, the weight function is defined as wi=e−tiτ

where *t*_*i*_ is the (positive) time interval between the R wave corresponding to the QT interval and the end of RR_i_, and τ is the time-constant of the model. The immediate response value providing the best fit was selected in each subject for each model.

4/ in addition to the models described above, we evaluated 2 models previously reported in the AF literature. These included a model based on average RR over the preceding 15 s (labeled **M15**) [19] and the model proposed by Ehlert et al. [20] and used by Larroude et al. [21] (i.e. $RRc=\frac{5*{RR}_{0}+2*{RR}_{-1}+{RR}_{-2}+{RR}_{-3}+{RR}_{-4}}{10}$; labeled **Mehl**).

Examples of weight functions are shown in **S2 Fig**. The formal description of the individual models is provided in **Table 1**.

**Table 1:** Formal description of individual models.

Model	Immediate Response	*w*_*i*_
**M0**	1	-
**M15**	0	1 if *t*_*i*_ < 15 s; 0 otherwise
**Mehl**	0.5	*w*_*-1*_ = 4; *w*_*-2*_ = *w*_*-3*_ = *w*_*-4*_ = 2; *w*_*i*_ = 0 otherwise
**M30exp**	Individually optimized in each subject	wi=e−ti30 if *t*_*i*_ < 180 s; 0 otherwise
**M60exp**	Individually optimized in each subject	wi=e−ti60 if *t*_*i*_ < 180 s; 0 otherwise
**M120exp**	Individually optimized in each subject	wi=e−ti120 if *t*_*i*_ < 180 s; 0 otherwise
**Mpopul**	0.183	wi=e−ti120 if *t*_*i*_ < 180 s; 0 otherwise
**Mpopul0**	0	wi=e−ti120 if *t*_*i*_ < 180 s; 0 otherwise

The models are defined by Eq 1 with the parameters listed in the Table. For the **M0** model, RRc = RR_0_, implying IR = 1; the second term in Eq 1 equals zero. The *t*_*i*_ values are expressed in seconds.

5/ for each subject, the fit of a particular model was then calculated using the natural logarithm of the ratio of the sum of squares provided by differences between the raw ST-T signals and the signal-averaged ST-T complex within each bin of the model based on RR_0_ only to the sum of squares based on the evaluated model, e.g.: ${fit}_{M15}=ln(\frac{{SSQ}_{M0}}{{SSQ}_{M15}})$.

6/ the QT interval was then determined for each of the 20 signal-averaged QRST complexes using an algorithm described and validated previously [22].

7/ finally, linear regression parameters were calculated for each subject from the 20 QT/RRc pairs. For the model **M0** (based only on RR_0_), the average corrected QT interval values based on Bazett and Fridericia formulas (QTcB and QTcF, respectively) as well as the slopes of QTcB/RR and QTcF/RR regression lines were calculated. The Bazett and Fridericia formulas (when RR_0_, QT and QTc are given in ms) are:

$$QTcB=QT.\sqrt{\frac{1000}{{RR}_{0}}}$$ (2)

and

$$QTcF=QT.\sqrt[{3}]{\frac{1000}{{RR}_{0}}}$$ (3)

A simplified flowchart summarizing the data analysis algorithm is provided in **S3 Fig.**

### Statistical analysis

The data are expressed as average ± standard deviation unless stated otherwise. The data analysis was performed with the statistical module of Excel 2013. Paired t-test was used to compare performance of individual models. All listed p values are 2-tailed; p<0.05 was considered statistically significant. No formal correction for multiple comparisons was performed. Wilcoxon test was used to compare squares of differences between measured QT values and QT values predicted by Bazett, Fridericia and **Mpopul**.

## Results

### Demographics

Data from 30 patients (69.3±7.3 years, 29 men) were analyzed. Left ventricular ejection fraction (LVEF) assessment was unavailable in 2 patients; LVEF was < 50% on the most recent assessment available prior to Holter monitoring in 4 (14%) patients. Coronary artery disease was present in 7 (23%), hypertension in 22 (73%), diabetes mellitus in 9 (30%), chronic kidney disease in 1 (3%), and obstructive sleep apnea in 5 patients (17%). Beta-blockers were used by 26 patients at the time of the recording; 2 of these were using sotalol. No other cardiac medications associated with QT prolongation were used. The average number of beats analyzed in a subject was 51,035±28,653; the RR interval preceding the analyzed beats was 819±215 ms (see also **S4 Fig**). Information regarding AF burden can be found in S1 Text.

### Comparison of model fits

**Fig 1:**
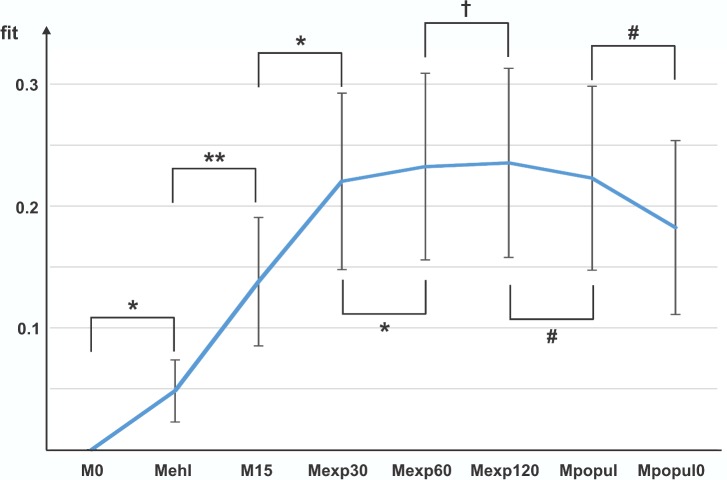
Comparison of data fit among discussed models. The fit of the **M0** model, which is used as a reference, is zero by definition. The vertical bars indicate 95% confidence intervals for the fit means. The symbols indicate statistical significance of the pairwise comparisons with paired t-test: * p<0.001; ** p<0.0001; **#** p<0.01, **†** p<0.05.

The model comparison indicated that the models can be ranked from worst to best in the sequence **M0** < **Mehl** < **M15** < **Mexp30** < **Mexp60** < **Mexp120.** All steps were statistically significant. The population-based model **Mpopul** (derived from **Mexp120** but with the immediate response fixed) was inferior to **Mexp120** as expected, but still performed substantially better than **M15** (p<0.0005). Elimination of immediate response from **Mpopul** (setting IR to 0; **Mpopul0**), resulted in a significant decrease in model fit (p<0.002). This confirms the important role of the immediate response component in the description of QT behavior. The results are summarized in **Fig 1**. Examples of data fit with **M0** and **Mexp120** from 2 subjects are provided in **S5 Fig**.

### Comparison of QTc estimates

The Bazett formula provided significantly longer average QTc estimates than the Fridericia formula (419±31 vs 401±33 ms; p<0.0001). The QTc calculated from the **Mexp120** model was 408±28 ms, significantly shorter than QTcB (p<0.01) and longer than QTcF (p<0.05).

### Population-level assessment of QT/RR dependence

The approach outlined above identifies the best model of QT dependence on HR in each individual subject. However, it would be desirable to derive a population-based formula comparable to Bazett or Fridericia (both of which only use a single free parameter, namely QTc, to describe QT/RR dependence), which could be used without prior evaluation of the best model in each patient. In order to accomplish this, we used the model providing the best fit across the study population (namely **Mexp120**; see **Results**section) with the average value of immediate response weight and the average value of the QT/RRc slope. In contrast to **Mexp120**, this model (**Mpopul**) only has a single free parameter (QTc) and its performance can thus be directly compared to performance of the Bazett and Fridericia formulas. Specifically, the QTc (in ms) based on this model can be written as:

QTc=QT+0.191.(1000-RRc) (4)

where RRc is provided by Eq 1, with IR = 0.183 and τ = 120 s. The performance of this model (**Mpopul**) was compared with the other models described above using the binning and signal-averaging method.

### Comparison of different QTc formulas

Several approaches were used to compare performance of the population-based formulas derived from the above models (and corresponding QT correction formulas) to the established QTcB and QTcF formulas. First, signal averaging was used to compare the average QTc values provided by different formulas and to determine the slope of QTc/RR relationship (which should equal zero for a perfect correction formula).

Second, manual measurements of raw QT intervals were obtained to determine the spread of QTc estimates. This approach was used to assess the performance of the individual correction formulas without the use of signal averaging, simulating a “real-life” clinical setting with manual QT determination. The standard deviations of patient-specific QTc values derived from individual formulas were used to measure the spread of QTc calculated from manual measurements. Here, low standard deviation of QTc values indicates good QTc formula performance. Specifically, 20 QT intervals from good-quality beats in each of the 30 subjects were measured using electronic calipers and the tangent method. The first good quality QT interval of each hour was selected to minimize selection bias. If this did not provide 20 QT intervals, the first suitable QT interval was measured in each 30 min (or eventually each 15 min) segment of the Holter recording until 20 QT intervals were measured in each subject. The corresponding QTc values were calculated using the Bazett and Fridericia formulas and population-based formulas derived from the **Mehl**, **M15,** and **Mpopul** models. A stretch of 60 s of continuous QTc intervals was also measured in a similar manner from 1 patient for illustrative purposes. Bland-Altman plots were used to compare the manually measured QT values to QT values derived from various models (Bazett, Fridericia, **Mpopul**) using patient-specific QTc values.

The QTc estimate provided by the **Mpopul** model and fixed RR/QT slope of 0.191, i.e. QTc calculated from Eq 4, was 402±28 ms, significantly shorter than QTcB (p<0.0001) and the estimate provided by the **Mexp120** model (p<0.05), and similar to QTcF (NS).

**Fig 2:**
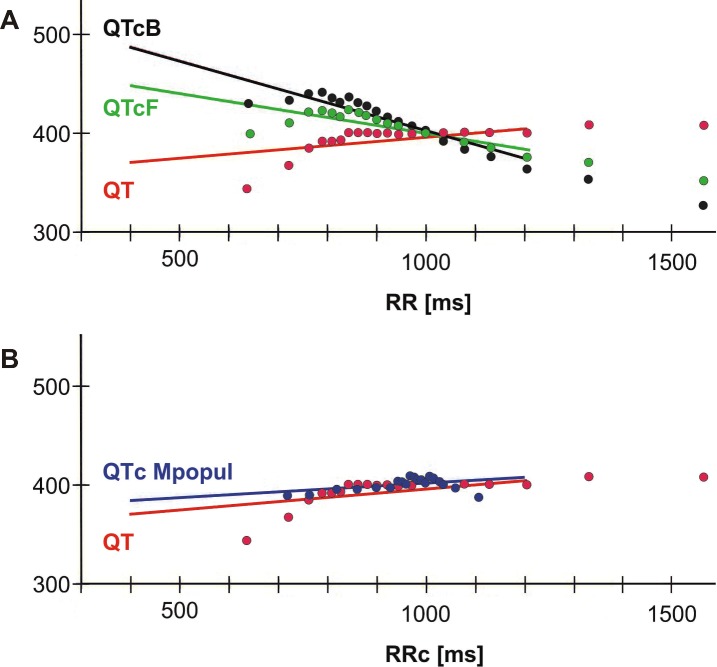
QTc/RR regression. **A** Data from a single patient derived from the **M0** model. In addition to the QT/RR data (**red**), corrected QT intervals based on Bazett (**black**) and Fridericia (**green**) formulas are displayed along with the corresponding QTc/RR regression lines. Note the markedly negative slope of both QTc regression lines, indicating poor performance of both formulas, namely overcorrection for fast heart rates. The regression lines are extended to a region without data points for clarity of labeling only. **B** QT/RR data are compared to QTc/RRc plot from the same patient derived from the **Mpopul** model. The slope of the regression line derived from **Mpopul** (**blue**) is smaller and the data fit is better than for QTcB and QTcF. Similar to the Bazett, Fridericia, and Framingham formulas, QTc derived from **Mpopul** has only one parameter (QTc). In contrast, the regression line slope and the IR parameter are also individually determined in the related **Mexp120** model.

The slopes of the QTcB/RR and QTcF/RR regression lines are both negative and significantly different from zero (-0.171±0.038 and -0.084±0.022 respectively; p<0.0001 in both cases), indicating overcorrection for fast HR. The slope is steeper (more negative) for QTcB than for QTcF (p<0.0001). The slope of the QTc/RR regression lines derived from **Mexp120** and the average regression slope derived from **Mpopul** are zero by definition. An example of regression lines derived from **M0** and **Mexp120** from a single patient is provided in **Fig 2**.

### QTc derived from manual QT measurements

The results of Bland-Altman analysis are summarized in **S6 Fig**. The squared differences between measured QT intervals and predicted QT values was highest for the Bazett model (1,133±2,290 ms^2^), intermediate for Fridericia (512±897 ms^2^) and best for **Mpopul** (216±546 ms^2^). The median of the squared differences was significantly higher for Bazett than for Fridericia and for Fridericia compared to **Mpopul** (p<0.001; Wilcoxon test).

**Fig 3:**
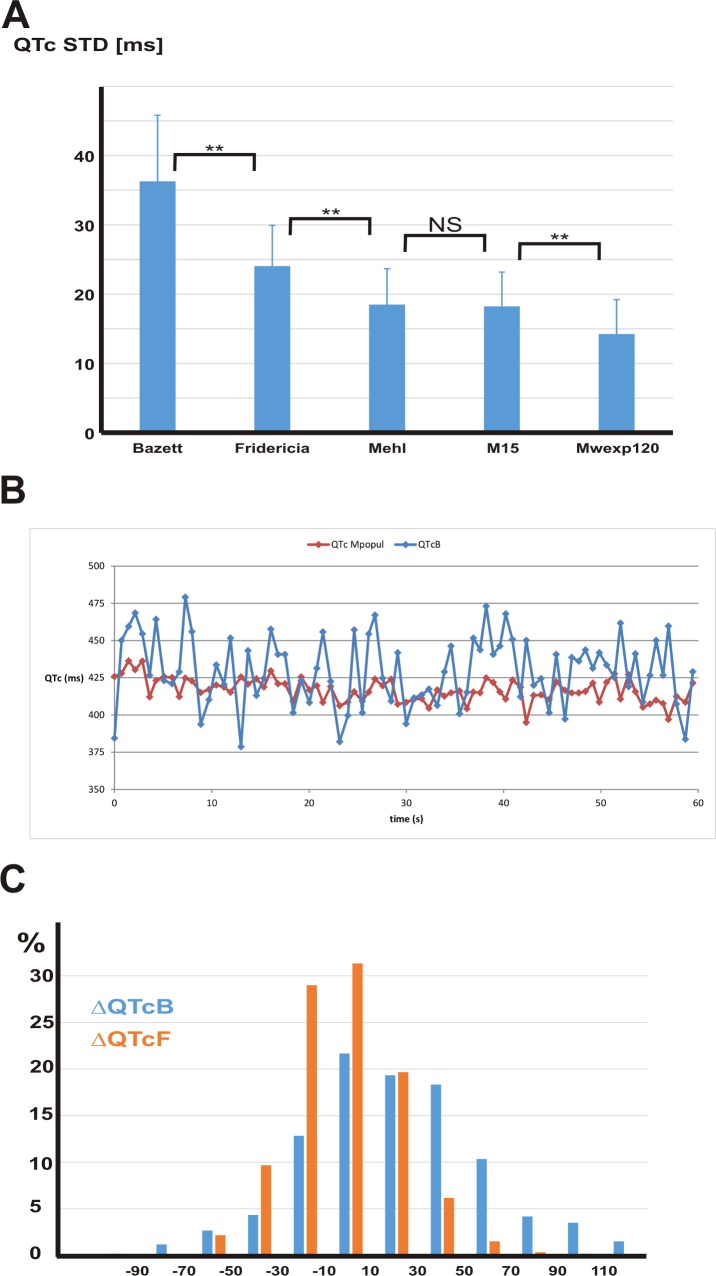
Comparison of QTc values. **A** The performance of individual correction formulas using standard deviation of 20 QTc values from each patient. The standard deviation is the highest for the Bazett formula and the lowest for the **Mpopul** formula. The error bars denote standard deviations. The symbol ** indicates p < 10^−4^. **B** QTc values from a 60 s recording from an individual patient demonstrating visually the difference in consistency between the **Mpopul** and Bazett corrections. **C** The distribution of differences between QTc values calculated by Bazett (**blue**) or Fridericia (**orange**) formulas from the QTc values calculated by the best model (**Mpopul**). The histogram is derived from 600 manually measured QT intervals (20 measurements in each patient). The QTcB and QTcF values differ from the best QTc estimate by > 30 ms in 54% and 20% of measurements, respectively. The data in this Figure indicate that the **Mpopul** formula performs better than the alternatives when manual QT measurement are used as the gold standard.

The consistency of QTc values obtained from the random manual measurements was the best for **Mpopul** (14.25±4.97 ms) and worst for the Bazett formula (36.28±9.53 ms); the other correction formulas provided intermediate values (**Fig 3A**). **Fig 3B** shows a 60 s stretch of QTc data calculated using the **Mpopul** and QTcB formulas. This is included for visual demonstration of the difference in consistency between the models. The distribution of QTcB and QTcF deviations from the values derived from the **Mpopul** model (using the random measurements) is shown in **Fig 3C**. It is apparent that a substantial proportion of QTc values derived by these formulas (54% and 20% respectively) differ from the optimal model by more than 30 ms despite the fact that they are derived from same QT measurements.

In order to place this in a clinical context, we calculated the number beats among the 600 with manual QT measurements in which QTcB provided values > 440 ms (the cutoff for initiation of dofetilide treatment), but the **Mpopul** model provided values < 440 ms. This occurred in 113/600 (18.8%) of beats. The opposite situation QTc < 440 ms using Bazett, but > 440 ms using **Mpopul** was present in 27/600 (4.5%) of beats. Overall, the use of **Mpopul** instead of Bazett formula would have changed the decision on dofetilide initiation in 23.3% of cases.

## Discussion

Reliable assessment of QT interval duration during AF is important. Repolarization delay predisposes patients to TdP and the short-long-short sequences that classically trigger TdP may be more common in AF than in SR. Many patients are treated for AF with class III antiarrhythmic drugs; these agents are often initiated while patients are in AF and reliable detection of excessive QT prolongation is necessary to minimize proarrhythmia [18,23].

The optimal way to correct QT interval for HR during AF is disputed. It is recognized that QT interval changes following the dramatic beat-to-beat changes in RR interval occurring in AF are substantially less pronounced than predicted by the Bazett formula, and it is known that Bazett formula overcorrects for the RR interval duration in AF [23] (as it does in SR [24,25]). Other QTc correction formulas are less prone to overcorrection, but it is uncertain how to best compare their performance since it is typically impossible to maintain steady HR at 60 bpm in most AF patients to determine the “correct” QTc. This situation is complicated by the superimposition of the fibrillatory wavelets and the terminal T wave portion, which can substantially decrease precision of a QT measurement in an individual beat.

In this study, we used signal-averaging to eliminate the superimposed atrial activity and tested several models of QT interval dependence on recent HR history including the formulas using the average RR interval over preceding 15 s [20] and the weighted average of 5 RR intervals preceding the QT interval proposed by Ehlert et al. [21] We find that the best data fit is provided by RR interval correction using an exponential weight function with a time-constant of 2 minutes combined with an immediate response to HR change with a weight of 15–20%. Interestingly, this is very similar to the QT interval response to sudden change in rate of atrial pacing we reported previously: the pacing data were well fit with an exponential weight function with time-constant of 60–90 s and immediate response with weight of 15% at rest and 47% during dobutamine infusion [10]. The slope of the steady-state QT/RR line obtained in this study (0.191) is also similar to that in SR and atrial pacing (approximately 0.2).

The slow QT response to HR changes during AF appears to be quite similar to SR as described in Holter data in normal subjects [11] and congenital LQTS patients [12]. In those papers, the exponential weight function with time constant of 60 s provided the best data fit, but longer time-constants were not studied. These published SR models did not incorporate the immediate response, which is probably much more important in AF than in SR because of higher beat-to-beat HR variability in AF. It is possible that a model combining a slow (time-constant 1–2 minutes) response with a small immediate response to HR change provides a fairly universal description of QT accommodation for AF, SR, and atrial pacing.

Our results are quite similar to the report of Pickham et al. [26], who used a more complex model of QT interval accommodation in AF (employing 8 independently optimized parameters). These authors also found a slope between the steady-state QT and RR of 0.17, comparable to the slope determined in our study. The magnitude of the immediate response (i.e. the **W0** parameter in the Pickham paper) is also similar to our result at approximately 0.17 (based on Fig.5 in reference [26]). Direct comparison of the two correction methods using the same dataset would be necessary to determine if one of the approaches is superior to another and if the apparently higher complexity of the Pickham method is justified.

This study indicates that appropriate HR correction of QT interval in AF may be difficult if only the 10 s of data typically stored in a standard 12-lead EKG is available since only ~25% of the overall HR effect on QT interval is incurred in this timeframe. In other words, a given value of QT interval may indicate either normal or delayed repolarization, depending on HR preceding it by more than 10 s, even if the RR interval sequence in the preceding 10 s was identical.

In principle, the population-based model **Mpopul** could be easily implemented in standard 12-lead EKG machines to improve the precision of QTc measurement during AF. This would require approximately 3 minute recordings if no signal-averaging is performed, and somewhat longer if signal-averaging is required to suppress the atrial signal and improve T end detection. Such approach could substantially improve detection of QT prolongation in AF, although its clinical utility remains to be determined. Consistent with the paper by Musat et al. [23], our data suggests that the QTc values obtained from Bazett formula in AF are too long (in addition to the undesirable HR dependence). Its use in AF may result in incorrect clinical decisions such as unnecessary cessation of treatment with dofetilide or sotalol.

In this study, we have also compared the performance of individual formulas without signal averaging, using manual QT interval measurements. The results confirm that even in this scenario, the **Mpopul** model provides the most consistent QTc values, which differ from the QTcB and QTcF values by > 30 ms–a value which can have a clinical impact—in a large subset of measurements. For example, our data indicate that the use of **Mpopu**l formula instead of Bazett could change the decision on dofetilide initiation in nearly a quarter of patients.

The molecular mechanisms accounting for the fast and slow QT interval accommodation components are uncertain. It is likely that the accumulation of the I_Ks_ open probability (time-constant measured in seconds) with HR increase accounts for the immediate response [27]. This is consistent with our previous report indicating augmentation of immediate response by β-adrenergic stimulation. The mechanisms responsible for the slow component of QT accommodation are uncertain and may involve changes in intracellular Ca^2+^ and Na^+^ concentration, affecting Ca^2+^-dependent I_CaL_ inactivation and I_NaK_ activity, respectively [28,29].

### Limitations

The data were collected retrospectively. Nearly all the patients were male, and most of them were not treated with antiarrhythmic drugs except for beta-blockers. Ideally, confirmation in an independent population with more diverse demographics studied prospectively should be performed. We could not analyze data during SR and AF from the same patients or compare patients with and without structural heart disease. We cannot prove that QT correction based on the approach outlined here is superior with respect to identification of patients who are at risk of TdP. However, such a proof would require a large number of patients with several minutes of EKG preceding TdP onset; this may not be a practical way for comparing QT correction formulas. Our data do indicate that the QTc formulas in current clinical use (QTcB and QTcF) deviate from the optimal correction by a large margin in a substantial proportion of measurements.

## Supporting information

S1 Fig An example of a holter tracing of acceptable quality. Triangles denote R wave peaks. RR intervals in ms are shown in **red**.
(TIF)

S2 Fig Examples of weight functions. **M15** model (**red**)–equal weight is assigned to all RR intervals preceding the QT interval by < 15 s. **Mpopul** model (**blue**)–a weight of 0.183 is assigned to the immediately preceding RR interval and the remaining weight (0.817) is distributed as an exponential function with 2 min time constant over the preceding 180 s. Both functions are scaled to provide area under the curve of 1.
(TIF)

S3 Fig Flowchart depicting a simplified version of the approach used for data analysis. (TIF)

S4 Fig Demographic data. Box-whisker plot showing means and interquartile ranges for age (years), mean RR interval in the analyzed data (ms), number of analyzed beats and left ventricular ejection fraction (**LVEF**; %) in the study population.
(TIF)

S5 Fig Comparison between M0 and Mexp120 Model fits. **A** Example signal-averaged QRST complexes from a single patient. **M0** model (**blue**); **Mexp120** model (**red**). Note that the T wave derived from the **M0** model has lower amplitude and is slightly wider at half-amplitude than the T wave from **Mexp120**, suggesting “smearing” of the T wave morphology caused by averaging T waves of slightly different shapes. Compared to **M0**, the **Mexp120** model captures more of the T wave variability in the form of difference between the signal-averaged signals corresponding to the individual heart rate bins, resulting in less variability within bins. **B** Signal-averaged QRST complexes derived from **M0** (left) and **Mexp120** (right) models. Every other ventile is skipped for clarity. Note the higher variability in morphology of T waves from different ventiles in the **Mexp120** model. This corresponds to less variability within bins and better model fit.
(TIF)

S6 Fig Bland-Altman comparisons. QT intervals measured manually (**QTm**) are compared with QT intervals derived from Bazett (**A**), Fridericia (**B**) and **Mpopul** (**C**) models (predicted QT intervals, **QTp**). In all panels, the average of predicted and measured QT values is plotted in the X-axis and their difference on the Y-axis. The **QTp** values are calculated from the respective formulas using patient-specific QTc values. The regression lines (**red**) and 95% confidence limits for the difference between measured and predicted QT values (**green**; based on assumption of normal distribution) are shown in each panel. The spread of differences between **QTm** and **QTp** is higher for Bazett than for Fridericia and lower for **Mpopul** than for Fridericia (p<0.001 for both comparisons), consistent with superior performance of **Mpopul**.
(TIF)

S1 Text AF burden demographics. (DOCX)

S1 Data Raw holter data part 1 of 6. (RAR)

S2 Data Raw holter data part 2 of 6. (RAR)

S3 Data Raw holter data part 3 of 6. (RAR)

S4 Data Raw holter data part 4 of 6. (RAR)

S5 Data Raw holter data part 5 of 6. (RAR)

S6 Data Raw holter data part 6 of 6. (RAR)
