# Supplementary figures and images for: Recent heart rate history affects QT interval duration in atrial fibrillation
Source: PLoS One. 2017 Mar 8;12(3):e0172962. doi: 10.1371/journal.pone.0172962 (PMC5342318; doi:10.1371/journal.pone.0172962)

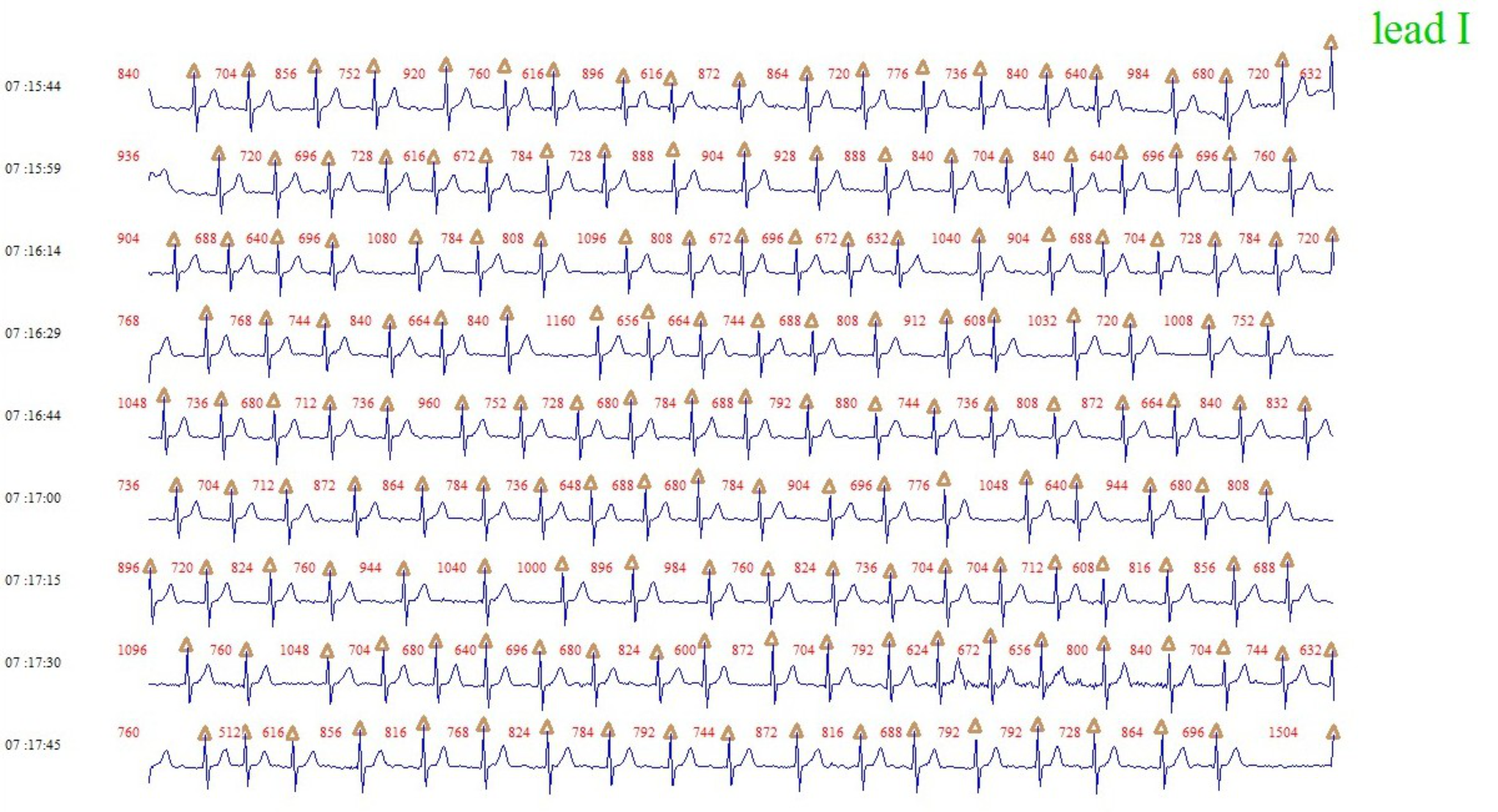

Supplement: S1 Fig — Triangles denote R wave peaks. RR intervals in ms are shown in red. (TIF) [file pone.0172962.s001.tif]

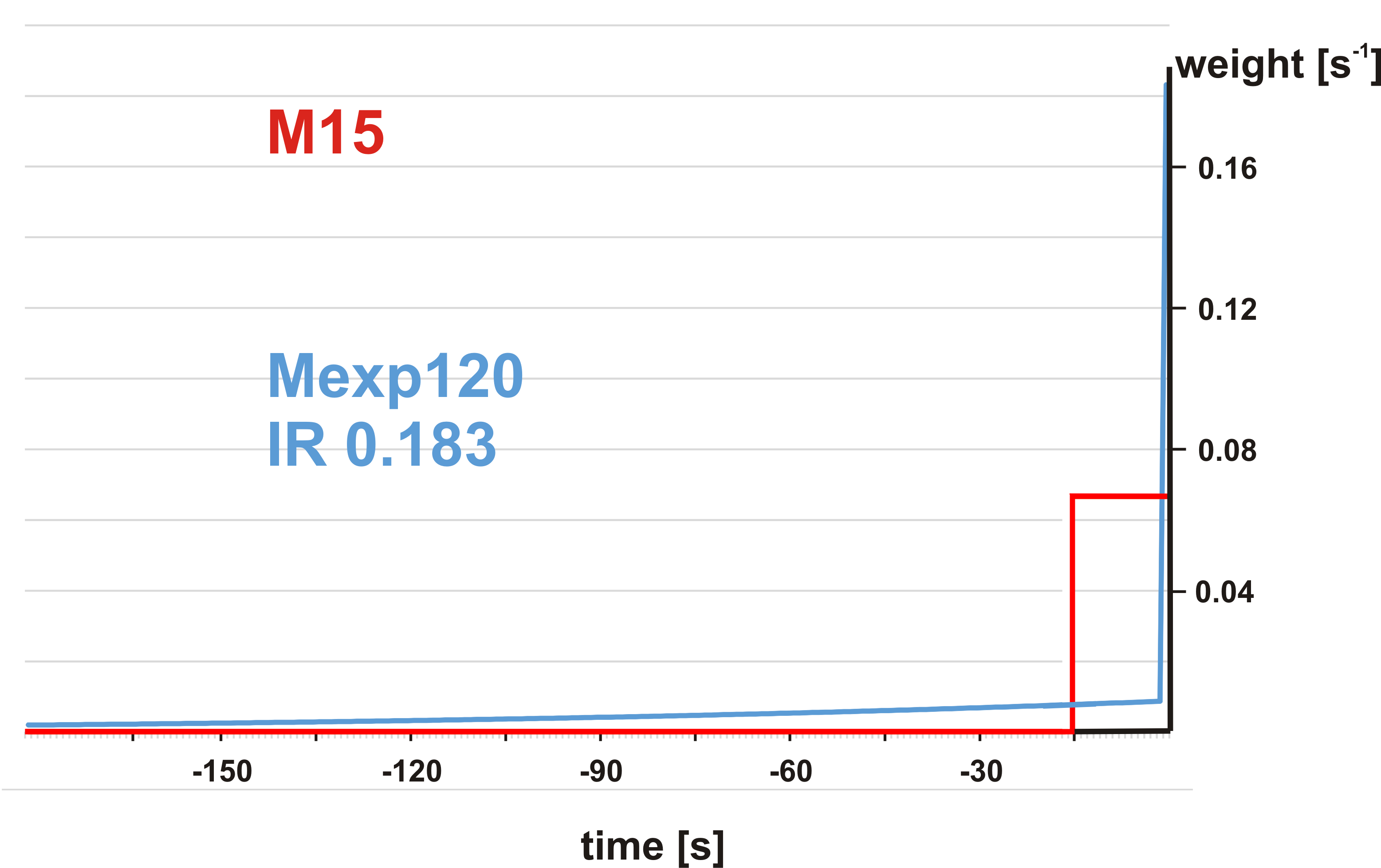

Supplement: S2 Fig — M15 model (red)–equal weight is assigned to all RR intervals preceding the QT interval by < 15 s. Mpopul model (blue)–a weight of 0.183 is assigned to the immediately preceding RR interval and the remaining weight (0.817) is distributed as an exponential function with 2 min time constant over the preceding 180 s. Both functions are scaled to provide area under the curve of 1. (TIF) [file pone.0172962.s002.tif]

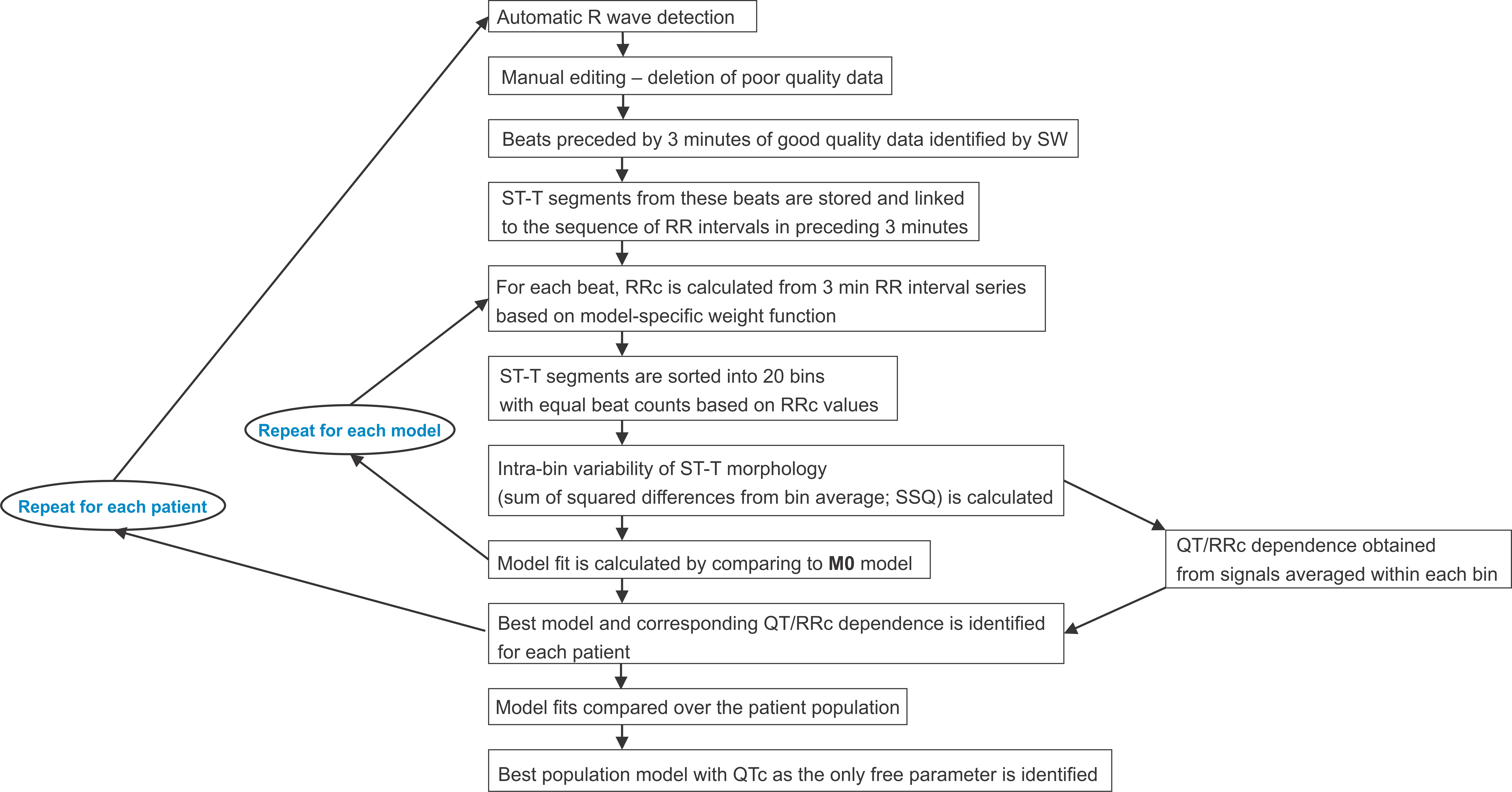

Supplement: S3 Fig — (TIF) [file pone.0172962.s003.tif]

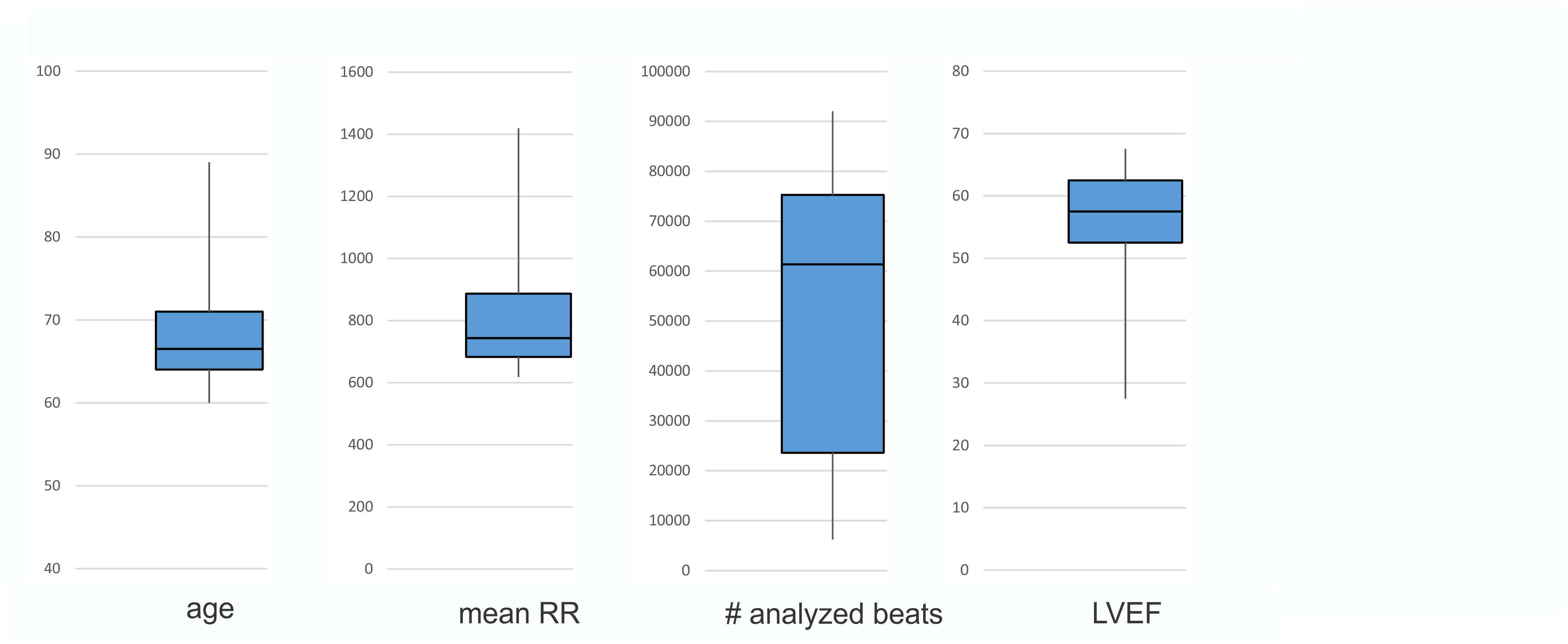

Supplement: S4 Fig — Box-whisker plot showing means and interquartile ranges for age (years), mean RR interval in the analyzed data (ms), number of analyzed beats and left ventricular ejection fraction (LVEF; %) in the study population. (TIF) [file pone.0172962.s004.tif]

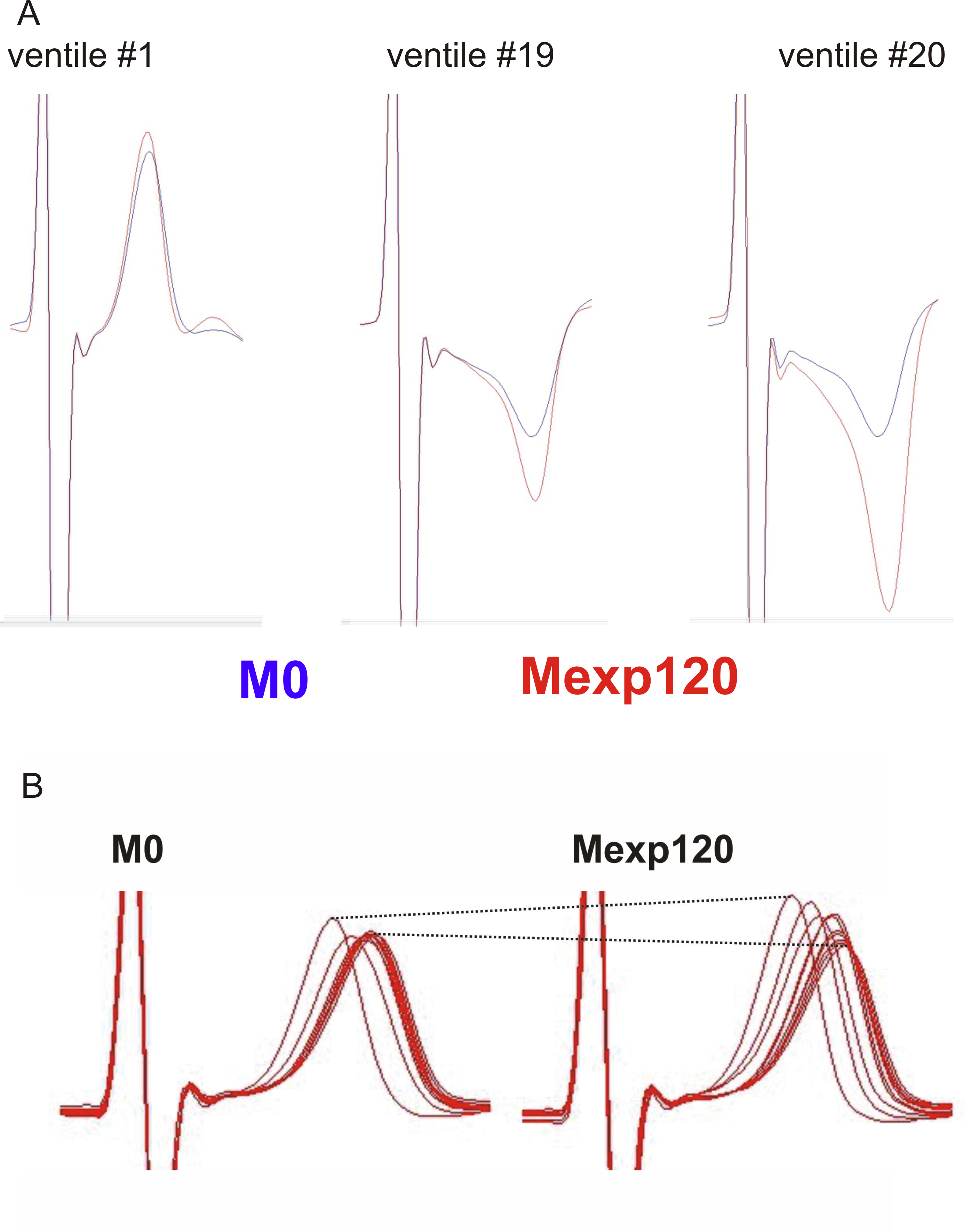

Supplement: S5 Fig — A Example signal-averaged QRST complexes from a single patient. M0 model (blue); Mexp120 model (red). Note that the T wave derived from the M0 model has lower amplitude and is slightly wider at half-amplitude than the T wave from Mexp120, suggesting “smearing” of the T wave morphology caused by averaging T waves of slightly different shapes. Compared to M0, the Mexp120 model captures more of the T wave variability in the form of difference between the signal-averaged signals corresponding to the individual heart rate bins, resulting in less variability within bins. B Signal-averaged QRST complexes derived from M0 (left) and Mexp120 (right) models. Every other ventile is skipped for clarity. Note the higher variability in morphology of T waves from different ventiles in the Mexp120 model. This corresponds to less variability within bins and better model fit. (TIF) [file pone.0172962.s005.tif]

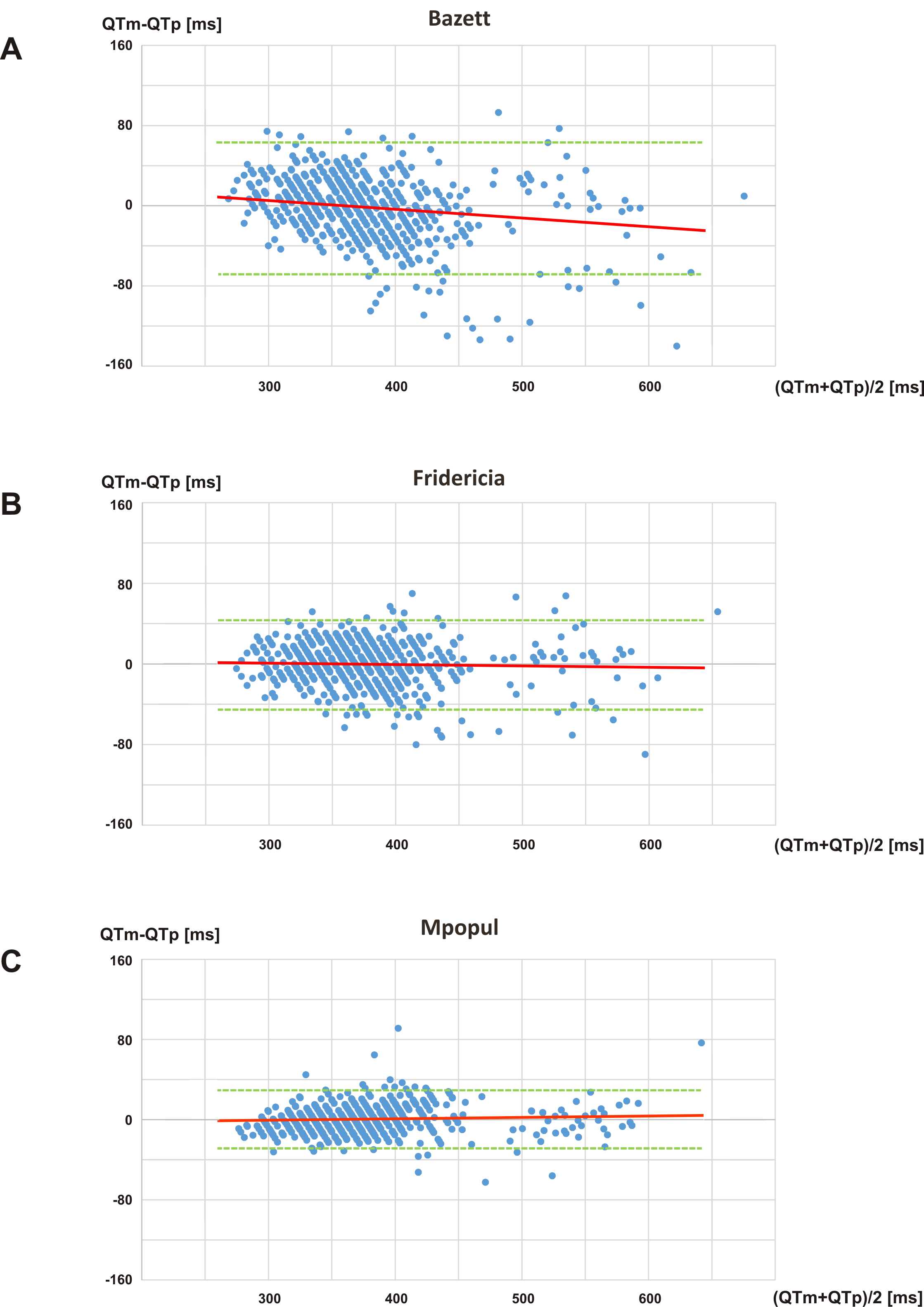

Supplement: S6 Fig — QT intervals measured manually (QTm) are compared with QT intervals derived from Bazett (A), Fridericia (B) and Mpopul (C) models (predicted QT intervals, QTp). In all panels, the average of predicted and measured QT values is plotted in the X-axis and their difference on the Y-axis. The QTp values are calculated from the respective formulas using patient-specific QTc values. The regression lines (red) and 95% confidence limits for the difference between measured and predicted QT values (green; based on assumption of normal distribution) are shown in each panel. The spread of differences between QTm and QTp is higher for Bazett than for Fridericia and lower for Mpopul than for Fridericia (p<0.001 for both comparisons), consistent with superior performance of Mpopul. (TIF) [file pone.0172962.s006.tif]
