# Supplementary material for: Recent heart rate history affects QT interval duration in atrial fibrillation
Source: PLoS One. 2017 Mar 8;12(3):e0172962. doi: 10.1371/journal.pone.0172962 (PMC5342318; doi:10.1371/journal.pone.0172962)
Supplement: S1 Text — (DOCX) [file pone.0172962.s007.docx]

**AF burden.** Nineteen patients (63%) had persistent AF, 9 (30%) had paroxysmal AF, and 2 (7%) had new onset AF at the time of holter recording. AF classification was determined by reviewing all holter and EKG data in MUSE prior to and including the index holter. Patients were classified as having persistent AF if all prior recordings demonstrated AF and paroxysmal AF if a combination of AF and non-AF rhythms were identified unless a clear time point before which all preceding rhythms were sinus or non-AF and all rhythms afterwards were AF. In this case patients, were classified as having persistent AF. If the index holter was the first evidence of AF in the patient’s chart, they were classified as having new onset AF.
